# The clinical burden of allergic rhinitis in five Middle Eastern countries: results of the SNAPSHOT program

**DOI:** 10.1186/s13223-018-0298-x

**Published:** 2018-11-19

**Authors:** Ahmed Al-Digheari, Bassam Mahboub, Hesham Tarraf, Taskin Yucel, Isabella Annesi-Maesano, Adam Doble, Aaicha Lahlou, Luqman Tariq, Fayaz Aziz, Abdelkader El Hasnaoui

**Affiliations:** 1 Department of Paediatrics, Allergy And Clinical Immunology, Security Forces Hospital, Ministry of Interior, Riyadh, Saudi Arabia; 2 College of Medicine, University of Sharjah, Sharjah, United Arab Emirates; 3 Pulmonary Medicine Department, Rashid Hospital, Dubai, United Arab Emirates; 4 The Medical School, Cairo University, Cairo, Egypt; 5 Department of Otorhinolaryngology, Hacettepe University, Ankara, Turkey; 6 Epidemiology of Allergic and Respiratory Diseases Department (EPAR), Institut Pierre Louis d’Epidemiologie et de Sante Publique (IPLESP), Sorbonne Universite and INSERM, Medical School Saint-Antoine, Paris, France; 7 Foxymed, Paris, France; 8 MS Health, Rabat, Morocco; 9 GlaxoSmithKline, PO Box 50199, Dubai, United Arab Emirates

**Keywords:** Allergic rhinitis, Clinical burden, Middle East, SNAPSHOT, Quality of life

## Abstract

**Background:**

The SNAPSHOT program provides current data on the allergic rhinitis burden in the adult general population of five Middle Eastern countries (Egypt, Turkey, Kuwait, Saudi Arabia and the United Arab Emirates, the latter three grouped into a Gulf cluster).

**Methods:**

A multi-country, cross-sectional, epidemiological program conducted by telephone in a random sample of the adult general population; quotas were defined per country demographics. Subjects were screened for allergic rhinitis using the Score For Allergic Rhinitis questionnaire. Current prevalence (last 12 months) was estimated. Disease severity and control were assessed using the Allergic Rhinitis and its Impact on Asthma classification and Rhinitis Control Assessment Test respectively. Quality of sleep, impact on daily activities and quality of life were measured using the Epworth Sleepiness Scale, Sheehan Disability Scale and EuroQol Five-Dimension questionnaire respectively. Multivariate logistic regression analyses were used to investigate risk factors and co-morbidities.

**Results:**

1808 of 33,486 subjects enrolled in the SNAPSHOT program fulfilled the case definition for allergic rhinitis. Prevalence was 3.6% [95% CI 3.2–4.0%] in Egypt, 6.4% [95% CI 5.9–6.9%] in Turkey and 6.4% [95% CI 6.0–6.9%] in the Gulf cluster. Risk factors identified were country, co-morbid asthma and income. Subjects with allergic rhinitis reported a significantly lower quality of life compared to the general population (*p *< 0.0001). Overall, 55% of allergic rhinitis subjects were moderate/severe and 33% were uncontrolled. Both these groups reported impaired quality of life and quality of sleep and increased impairment of daily activities compared to mild/well-controlled subjects (*p *< 0.0001).

**Conclusions:**

Although the observed prevalence of allergic rhinitis in these Middle Eastern countries is low compared to western countries, its burden is considerable. Allergic rhinitis in general, and specifically uncontrolled and severe disease, results in a negative impact on quality of life, quality of sleep and daily activities.

## Background

Allergic Rhinitis (AR) is a common inflammatory disorder affecting the nasal membranes characterised by symptoms such as sneezing and nasal congestion. It is a global health problem; conservative estimates suggest that approximately 500 million people worldwide are affected by the disease. The burden of AR is often under-estimated since the disease is not life-threatening. However, it can cause significant morbidity, through both the physical symptoms and its impact on quality of life and well-being [1]. It has a profound effect on many aspects of daily life such as sleep [2], work/school [3], and social life, having been shown to result in fatigue and mood changes [4], to impair cognitive function [5], and to lead to depression and anxiety [6].

Large scale multinational studies that have collected data on the prevalence of AR in adults are rare and those that have taken place use different study methodologies and case definitions, and report a wide range of prevalence estimates. Several studies have been conducted in Western Europe and the United States (US). For example, one study carried out in six European countries (Belgium, France, Germany, Italy, Spain and the United Kingdom (UK)) using the Allergic Rhinitis and its Impact on Asthma (ARIA) definition of AR reported that overall, the prevalence of AR, confirmed by clinical examination was 22.7%. However, the prevalence varied between countries from 16.9% in Italy to 28.5% in Belgium [7]. Data from the Burden of Rhinitis in America study reported the prevalence of rhinitis associated with seasonal or perennial rhinitis to range between 11.9% and 30.2% depending on duration of symptoms and physician diagnosis [8].

Data on the prevalence and burden of AR outside of these areas, and in the Middle East particularly, is scarce. Most studies have been carried out among school children, many of which have been based on the International Study of Asthma and Allergies in Childhood (ISAAC) questionnaires and a wide variation in prevalence has been reported. Differences in prevalence have been reported between countries in the region and prevalence has also been shown to vary between the age groups of the children included in the studies. For example, a nationwide survey in Oman reported a 7.4% prevalence of AR in 6–7 year olds and a 10.5% prevalence in 13–14 year olds, whereas a study in Qatar reported a 30.5% prevalence of physician diagnosed AR in children between 6 and 14 years old. Studies in adults have been carried out in the United Arab Emirates (UAE) [9], and Turkey [10–13] and the Allergies In the Middle East Survey (AIMES) reported the prevalence of AR in adults across five Middle Eastern countries (Egypt, Iran, Lebanon, Saudi Arabia and the UAE). However, once again, these studies use varying case definitions and a range of study designs making it difficult to compare the results.

To address the need for data in the Middle East on the prevalence and burden of AR, the SNAPSHOT program was conducted; a large, cross-sectional, population-based program using a standardised methodology in five countries in the region (Egypt, Kuwait, Saudi Arabia, Turkey, and the UAE), to investigate the current prevalence and disease burden attributable to AR in the adult population of these five countries.

## Methods

### The SNAPSHOT Program

SNAPSHOT is a multi-national, cross-sectional, population-based program, comprising multiple studies, conducted in a random sample of the adult general population of five countries (Egypt, Kuwait, Saudi Arabia, Turkey, and the UAE), between July 2014 and February 2016. The objective of the SNAPSHOT program was to provide an omnibus approach to collect data simultaneously about multiple diseases using a standardised methodology. The program provides updated epidemiological data on the prevalence, burden of disease, quality of life and healthcare resource use related to four chronic diseases in the participating countries: asthma, AR, bipolar disorder and benign prostatic hyperplasia (BPH). The selection of these diseases was based on the need to respond to emerging health technology assessment (HTA) requirements by local authorities in these Middle Eastern countries. The complete methodology and program rationale have been described in detail elsewhere [14].

A quota of 10,000 subjects from the adult general population of Turkey and Egypt and 15,000 from the Gulf cluster (comprised of Kuwait, Saudi Arabia and the UAE) was targeted based on the demographic structure of the country in terms of age and gender, using a random stratified sampling method. In the Gulf cluster, it was difficult to reach the target sample size, and since the combined number of interviews conducted to date in Saudi Arabia, Kuwait and the UAE showed that the program objectives could be achieved from a sample size perspective, recruitment was stopped in February 2016 and the database locked on the 11th of April 2016.

Subjects first responded to a screening questionnaire to identify subjects fulfilling the criteria for each of the diseases and record social and demographic characteristics, as well as the presence of co-morbidities. Respondents who met the criteria for one or more diseases were invited to continue the interview by replying to a detailed, disease-specific questionnaire. This questionnaire collected additional information on burden of disease, disease management and healthcare resource utilisation. Data on quality of life were also captured using the three level EuroQoL Five-Dimension questionnaire (EQ-5D-3L) (© EuroQol Research Foundation. EQ-5D™ is a trade mark of the EuroQol Research Foundation) [15, 16].

### Case definition

Subjects were screened for AR based on the Score For Allergic Rhinitis (SFAR) questionnaire, a validated screening tool which consists of 10 items with a score ranging from 0 to 16 [17]. The full questionnaire is shown in Table 1, together with the scores attributable to each item. A subject who scored a cumulative score greater than or equal to seven was classified as having AR. The SFAR questionnaire was presented in the language of the country where the study took place. The translated versions of the SFAR questionnaire in all three languages of the SNAPSHOT program were provided for use by the author and copyright holder of the questionnaire. Validation of the SFAR questionnaire can also be found in the literature [17], and translated versions have been previously used in Turkey [13], and the Maghreb [18].

**Table 1:** Score For Allergic Rhinitis (SFAR) questionnaire

Item	Options	Score	Total score
Q1. In the past 12 months, have you had a problem apart from cold or flu with: Sneezing? Runny nose? Blocked nose?	□ No □ Yes	Min 0, Max 3; 1 per symptom	3
If ‘NO’ do not respond to the following questions			
Q2. In the past 12 months has this nose problem been accompanied by itchy/watery eyes?	□ No □ Yes	Min 0, Max 2; YES = 2	5
Q3. In which of the past 12 months (or in which season) did this nose problem occur?	□ Jan □ Feb □ Mar □ Apr □ May □ Jun □ Jul □ Aug □ Sep □ Oct □ Nov □ Dec	Min 0, Max 2; 1 if ≥ 7 months; 1 for pollen season^a^	7
Q4. In the past 12 months has your nose problem disturbed your daily activity?	□ Never □ Sometimes □ Rarely □ Often	n/a	7
Q5. What trigger factors provoke or increase your nose problem?	□ No □ Yes	Min 0, Max 2; Yes to epithelia (animals) OR moulds only = 1; Yes to pollens or dust = 2; Yes to pollens or dust + epithelia or moulds = 2	9
House dust/dust mites? Pollens? Animals (cats, dogs etc.) Others?	□ No □ Yes □ No □ Yes □ No □ Yes		
Q6. Do you think you are allergic?	□ No □ Yes	Min 0, Max 2; YES = 2	11
Q7. Have you already been tested for allergy (SPT, IgE etc.)?	□ No □ Yes	Min 0, Max 2; Both YES = 2	13
If yes, was it positive?	□ No □ Yes		
Q8. Has a doctor already diagnosed that you suffer/suffered from an allergy (asthma, eczema, AR)?	□ No □ Yes	Min 0, Max 1; YES = 1	14
Q9. Do you think you are asthmatic?	□ No □ Yes	n/a	14
Q10. Does any member of your family suffer from asthma, eczema or AR?	□ Father □ Mother □ Sibling	Min 0, Max 2; ≥1 specified family member suffering from one allergy = 2	16

The score for allergic questionnaire, consists of 10 questionnaires, scored as indicated in the table. A subject who scored greater than or equal to seven was classified as having allergic rhinitis (AR)

^a^ Pollen season in the Middle East is defined as April, May, September and October [19]

### Data collected for this analysis

The analysis presented here focuses solely on AR and aims to provide estimates of the current prevalence of AR and demonstrate the burden of disease in the countries studied. AR prevalence data were collected using the case definition described and adjusted by age, gender and country. Socio-demographic data were collected to describe the characteristics of the overall program population, including gender and age distribution, height and weight (body mass index (BMI) was calculated), the presence of co-morbid conditions, family history of the disease and smoking status. If a respondent was a current or former smoker, they were asked to provide the duration and extent of exposure. Additionally, the type and frequency of co-morbidities was investigated and risk factors for AR were identified. As part of the screening questionnaire, subjects were also screened for asthma using a case definition based on the global Asthma Insights and Reality (AIR) surveys [20].

Control of AR was assessed using the Rhinitis Control Assessment Test (RCAT), a six item self-report questionnaire with responses on a five-point scale [21]. Symptom control over the past week was assessed; a score ≤ 21 indicates that rhinitis symptoms are not well-controlled and a score > 21 indicates that rhinitis symptoms are well controlled. Severity of the disease was assessed based on the 2008 ARIA classification, with symptoms being classed as mild or moderate/severe depending on the severity of symptoms and their impact on social life and school/work [1].

The level of daytime sleepiness was measured using the Epworth Sleepiness Scale (ESS), which is an eight-item self-report questionnaire, where respondents are asked to rate their usual chance of falling asleep while engaged in eight different activities on a four-point scale (0–3) [22]. A total score between zero and five indicates lower normal daytime sleepiness; between six and ten denotes higher normal daytime sleepiness; between 11 and 12 indicates mild excessive daytime sleepiness; between 13 and 15 indicates moderate excessive daytime sleepiness; and between 16 and 24 indicates excessive daytime sleepiness.

Daily activities were assessed using a modified version of the Sheehan Disability Scale (SDS), a patient reported outcome measure for assessing impairment of functioning in three major daily activities (work/school life; social life/leisure activities and family life/home responsibilities). In this study, the standard response options metric was modified to scoring on a 5-point Likert scale to accommodate data capture during a telephone interview. The response options used were ‘not at all/mildly/moderately/markedly/extremely’. During the analysis of the results, a ‘not at all’ was classified as ‘no’ and a response of ‘mildly/moderately/markedly/extremely’ was classified as ‘yes’. In this study, the timeframe used to assess symptom impact was “over the past 12 months”. (The SDS was used with the permission of the SDS copyright holder Professor DV Sheehan. © Sheehan DV 1996 & 2008 & 2016) [23, 24].

All subjects were also asked to complete the EQ-5D-3L questionnaire to measure quality of life, a generic questionnaire designed as a measure of health status which aims to capture the impact of a disease on physical, mental, and social functioning. The questionnaire consists of five dimensions (mobility, self-care, usual activities, pain/discomfort, anxiety/depression) each of which can take one of three responses (no problems/moderate problems/extreme problems) and a visual analogue rating scale (EQ-VAS) [15, 16].

All questionnaires were used in their validated English, Turkish or Arabic versions.

### Statistical analysis

Data are presented as proportions and means with standard deviations (SD), and 95% confidence intervals (95% CI) were calculated for binomial data. Associations between variables were estimated using the χ^2^ test and the  Kruskal–Wallis test as appropriate. Two-sided tests were used in all cases and a probability threshold of 0.05 was considered significant. Multivariate regression analysis was performed to identify the risk factors associated with AR. In the first step, specified variables were evaluated independently in a univariate analysis. All variables with a *p* value < 0.20 [25] in the univariate analysis were entered into the multiple logistic regression analysis. Variables were retained in the model using a backward selection to identify those associated with an increased risk of AR at the probability level of 0.05. A final multivariate analysis was performed to generate odds ratios. Multivariate regression analysis was also performed to assess the relationship between co-morbidities and AR independent of age, gender and country. All statistical analyses were performed using SAS^®^ Version 9.4 (SAS, Cary, USA).

## Results

### Study sample

A total of 33,486 subjects agreed to participate in the program, completed all the screening questionnaires and thus constituted the screening population. This population was distributed between Egypt (10,014), Turkey (10,000) and the Gulf cluster (13,472). Of the subjects enrolled in the program, 1808 fulfilled the case definition for AR and were defined as the AR population. This was the population used to calculate the prevalence of AR and investigate the risk factors, association with co-morbidities and impact on quality of life. Of these 1808 subjects, 857 (47.4%) completed the entire detailed AR questionnaire and constituted the AR responders used to investigate the burden of disease. This included an assessment of the severity and control of AR reported by the subjects and the impact on activities of daily living, quality of life and daytime sleepiness.

Selected demographics of the SNAPSHOT population and those subjects with AR are shown in Table 2. Demographically, there were few major differences between the 857 subjects with AR that completed the detailed questionnaire and the 1808 subjects who constituted the total AR population. The distribution in terms of age, gender, BMI, and smoking status is similar between the two populations (overall AR population and AR responders). The main difference between these two groups appears to be in health system coverage. A higher proportion of subjects in the AR responders group are covered by social security and a lower proportion are uninsured.

**Table 2:** Demographics

Demographics	Screening population N = 33,486	AR population N = 1808	AR non-responders N = 951	AR responders N = 857	AR responders		
					Egypt N = 145	Gulf cluster N = 234	Turkey N = 478
Gender n (%)							
Count	33,486	1808	951	857	145	234	478
Men	19,610 (58.6)	704 (38.9)	413 (43.4)	291 (34.0)	49 (33.8)	106 (45.3)	136 (28.5)
Women	13,876 (41.4)	1104 (61.1)	538 (56.6)	566 (66.0)	96 (66.2)	128 (54.7)	342 (71.5)
Age (years) n (%)							
Count	33,486	1808	951	857	145	234	478
18–34	15,959 (47.7)	856 (47.3)	452 (47.5)	404 (47.1)	83 (57.2)	129 (55.1)	192 (40.2)
35–49	9921 (29.6)	579 (32.0)	296 (31.1)	283 (33.0)	40 (27.6)	83 (35.5)	160 (33.5)
≥ 50	7606 (22.7)	373 (20.6)	203 (21.3)	170 (19.8)	22 (15.2)	22 (9.4)	126 (26.4)
Smoking n (%)							
Count	32,612	1771	918	853	145	234	474
Non-smoker	21,631 (66.3)	1238 (66.9)	650 (70.8)	588 (68.9)	106 (73.1)	162 (69.2)	320 (67.5)
Current/former smoker	10,981 (33.7)	533 (30.1)	268 (29.2)	265 (31.1)	39 (26.9)	72 (30.8)	154 (32.5)
BMI (Kg/m^2^) n (%)							
Count	30,522	1700	862	838	143	232	463
Underweight	970 (3.2)	62 (3.6)	34 (3.9)	28 (3.3)	3 (2.1)	10 (4.3)	15 (3.2)
Normal weight	11,491 (37.7)	629 (37.0)	286 (33.2)	343 (40.9)	47 (32.9)	80 (34.5)	216 (46.7)
Overweight	10,885 (35.7)	562 (33.1)	299 (34.7)	263 (31.4)	36 (25.2)	72 (31.0)	155 (33.5)
Obese	7176 (23.5)	447 (26.3)	243 (28.2)	204 (24.3)	57 (39.9)	70 (30.2)	77 (16.6)
Health system coverage n (%)							
Count	31,672	1742	892	850	143	233	474
Public	4502 (14.2)	206 (11.8)	140 (15.7)	66 (7.8)	24 (16.8)	40 (17.2)	2 (0.4)
Private/insured	5224 (16.5)	304 (17.5)	206 (23.1)	98 (11.5)	12 (8.4)	68 (29.2)	18 (3.8)
Social security	8350 (26.4)	536 (30.8)	118 (13.2)	418 (49.2)	0.0	2 (0.9)	416 (87.8)
Personal finances	1187 (3.8)	66 (3.8)	45 (5.0)	21 (2.5)	7 (4.9)	14 (6.0)	0.0
Not insured	12,341 (39.0)	629 (36.1)	382 (42.8)	247 (29.1)	100 (69.9)	109 (46.8)	38 (8.0)
Other	68 (0.2)	1 (0.1)	1 (0.1)	0	0	0	0

The demographics of the overall screening population (N = 33,486), the identified allergic rhinitis (AR) population (n = 1808), comparing the non-responders (n = 951) and responders (n = 857) to the detailed questionnaire; and the AR responders used to study the burden of disease by country or cluster studied

*BMI* body mass index

### Current prevalence of allergic rhinitis

Overall, the adjusted prevalence of AR in the adult general population over 18 years of age in the countries studied was 5.6% [95% CI 5.3–5.8]. This ranged from 3.6% [95% CI 3.2–4.0] in Egypt, to 6.4% [95% CI 5.9–6.9] in Turkey and 6.4% [95% CI 6.0–6.9] in the Gulf cluster. After adjusting for age, gender and country there is little difference between the crude and adjusted prevalence. These results are presented in Table 3.

**Table 3:** Current prevalence of allergic rhinitis

Variable	Overall N = 33,486	Egypt n = 10,014	Gulf cluster n = 13,472	Turkey n = 10,000
Number of cases	1808	343	845	620
Crude prevalence (%)	5.4	3.4	6.3	6.2
Adjusted prevalence (%)	5.6	3.6	6.4	6.4
Adjusted 95% CI	[5.33; 5.82]	[3.23; 3.96]	[6.02; 6.85]	[5.91; 6.87]

Current prevalence (%) of allergic rhinitis: adjusted by country, age and gender as required

*95% CI* 95% confidence interval

### Risk factors for allergic rhinitis

**Fig. 1:**
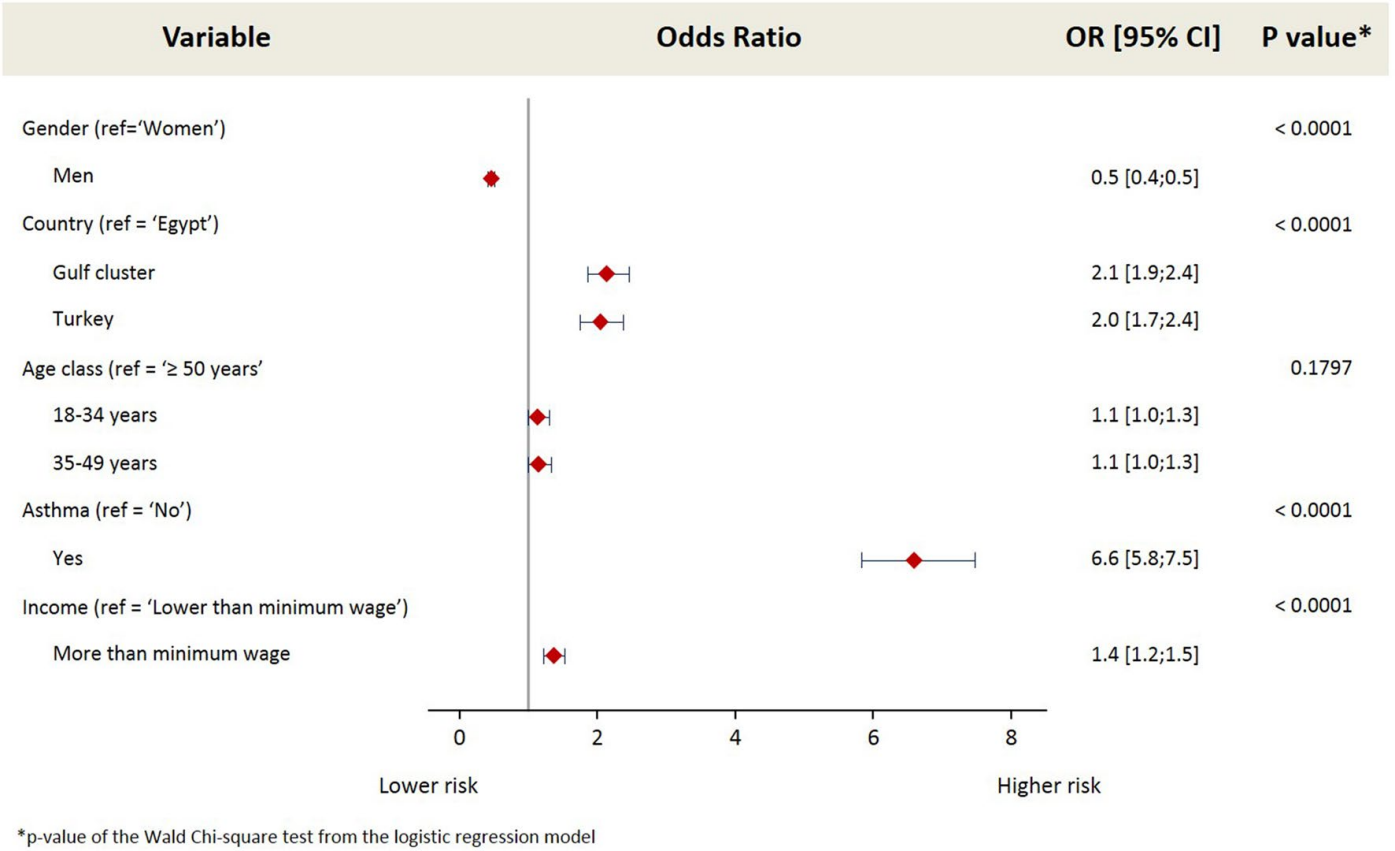
Risk factors for allergic rhinitis. Multivariate regression analysis investigating the risk factors for allergic rhinitis (AR): AR population (1679 subjects) versus non-AR population (28,351 subjects); OR [95% CI] = odds ratio [95% confidence interval]. The results presented are adjusted for age (18–34, 35–49, ≥ 50 years), gender and country (Egypt, Kuwait, Saudi Arabia, Turkey, UAE)

The following variables were tested using univariate analysis and found to have a significant association (*p *< 0.20) with AR: gender, age, country, health system coverage, income, BMI, having asthma or chronic obstructive pulmonary disease (COPD) and having a family history of AR. When these variables were entered into the multivariate analysis, a significant association was found between living in the Gulf cluster or Turkey and a higher risk of AR (odds ratio (OR) 2.1 and 2.0 respectively) compared to living in Egypt (*p *< 0.0001). In addition, a significant association (*p *< 0.0001) was observed between having asthma and a higher risk of AR (OR 6.6; 95% CI 5.8–7.5). Income was also shown to play a role; subjects with an income above the minimum wage were associated with a higher risk of AR (OR 1.4; 95% CI 1.2–1.5) compared to those with an income lower than the minimum wage. In addition, men were shown to have a lower risk of AR compared to women (OR 0.5; 95% CI 0.4–0.5) (*p *< 0.0001). These results are shown in Fig. 1.

### History of co-morbidities and the impact on the allergic rhinitis population

**Fig. 2:**
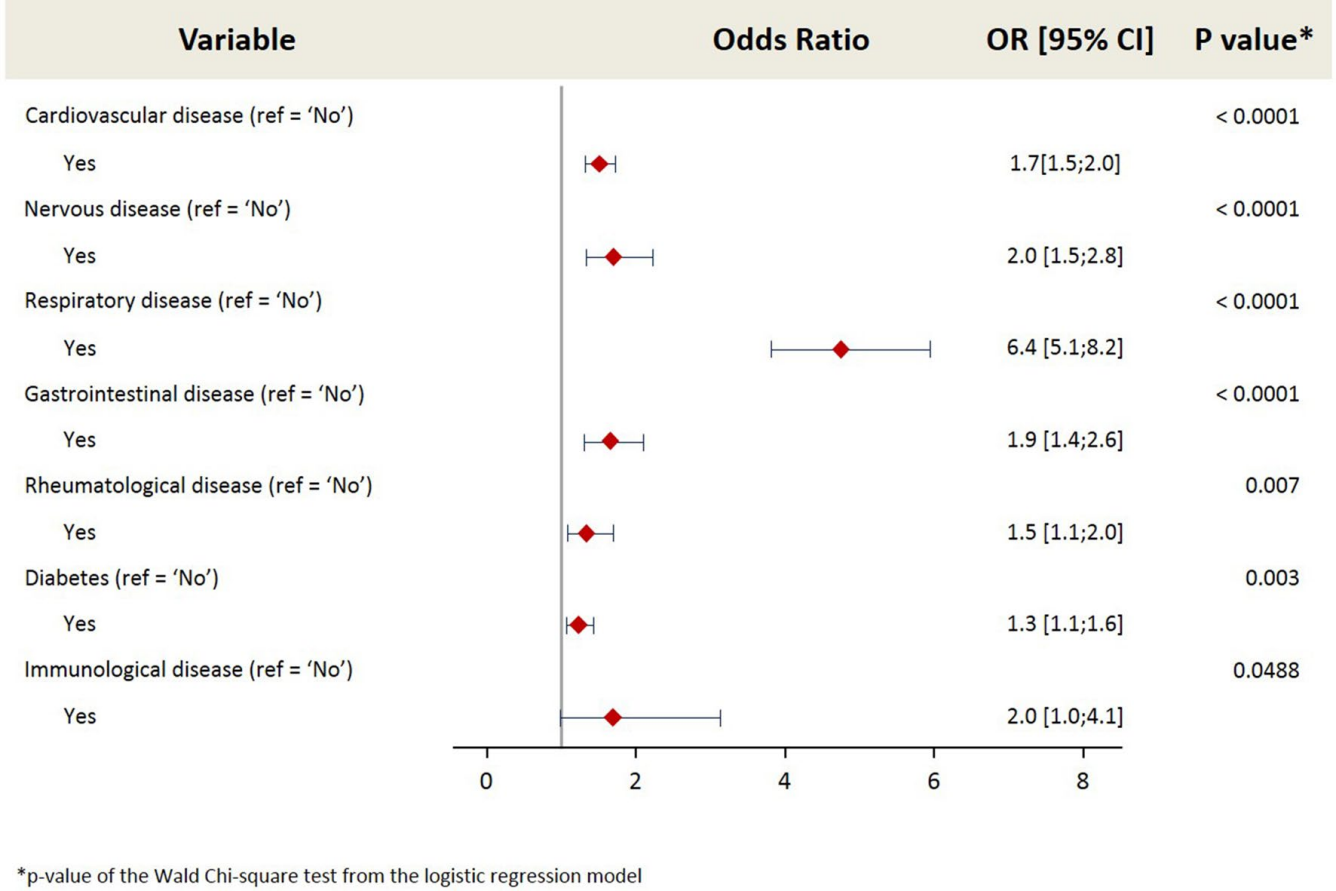
Impact of co-morbidities on the risk of allergic rhinitis. Multivariate regression analysis investigating the impact of co-morbidities. Allergic rhinitis (AR) population (1808 subjects) versus non-AR population (31,678 subjects); OR [95% CI] = odds ratio [95% confidence interval]. The results presented are adjusted for age (18–34, 35–49, ≥ 50 years), gender and country (Egypt, Kuwait, Saudi Arabia, Turkey, UAE)

The number of subjects that reported suffering from a chronic health condition was significantly higher (*p *< 0.0001) in the AR population (30.2%; n = 546) compared to the non-AR population (16.1%; n = 5104). The following co-morbidities were found to have a significant association (*p *< 0.20) with AR in a univariate analysis: cardiovascular, gastrointestinal, immunological, neurological, nervous, renal, respiratory, rheumatological diseases, diabetes and malignancy. These co-morbidities were entered into a multivariate analysis to investigate the relationship with AR, adjusting for age, gender and country. The co-morbidity with the highest impact was respiratory disease (OR 6.4). Detailed investigation into the conditions classified under the co-morbidity ‘respiratory disease’ revealed that this refers primarily to COPD. This was followed by nervous disease (OR 2.0), gastrointestinal disease (OR 1.9), cardiovascular disease (OR 1.7), rheumatological disease (OR 1.5), and diabetes (OR 1.3). These results are shown in Fig. 2.

### Characteristics of the allergic rhinitis responders

More than half the subjects with AR had moderate/severe disease (55.9%) as defined by the ARIA classification. However, the majority (67.0%) reported their symptoms as well-controlled (RCAT ≥ 21) and most (59.3%) reported low levels of daytime sleepiness (ESS 0–5). At a country level, significantly more patients reported mild disease in Turkey (59%; n = 282) compared to Egypt (23.4%; n = 34) and the Gulf cluster (26.5%; n = 62) (*p *< 0.0001); and Egypt reported a significantly lower proportion of subjects with well-controlled symptoms (44.4%; n = 52) compared to Turkey (72.1%; n = 313) and the Gulf cluster (69.5%; n = 121) (*p *< 0.0001). Overall, approximately 39% of subjects reported that AR symptoms had disrupted their work/school, social/leisure and family/home life. More subjects in Egypt reported disruption to their daily activities compared to the Gulf cluster and Turkey. These results are presented in Table 4.

**Table 4:** Characteristics of the allergic rhinitis (AR) population

Variable	Overall N = 857	Egypt n = 145	Gulf cluster n = 234	Turkey n = 478	*P* value
Severity (ARIA) N (%)					
Count	857	145	234	478	< 0.0001
Mild	378 (44.1)	34 (23.4)	62 (26.5)	282 (59.0)	
Moderate/severe	479 (55.9)	111 (76.6)	172 (73.5)	196 (41.0)	
Control (RCAT) N (%)					
Count	725	117	174	434	< 0.0001
≤ 21	239 (33.0)	65 (55.6)	53 (30.5)	121 (27.9)	
> 21	486 (67.0)	52 (44.4)	121 (69.5)	313 (72.1)	
ESS N (%)					
Count	720	115	173	422	< 0.0001
0–5	421 (59.3)	46 (40.0)	70 (40.5)	305 (72.3)	
6–10	157 (22.1)	50 (43.5)	66 (38.2)	41 (9.7)	
11–12	33 (4.6)	8 (7.0)	16 (9.2)	9 (2.1)	
13–15	29 (4.1)	4 (3.5)	11 (6.4)	14 (3.3)	
16–24	70 (9.9)	7 (6.1)	10 (5.8)	53 (12.6)	
SDS: Have your symptoms disrupted your work/school? N (%)					
Count	723	114	169	440	0.0881
No	441 (61.0)	59 (51.8)	106 (62.7)	276 (62.7)	
Yes	282 (39.0)	55 (48.2)	63 (37.3)	164 (37.3)	
SDS: Have your symptoms disrupted your social life/leisure activities? N (%)					
Count	722	113	169	440	0.2431
No	436 (60.4)	61 (54.0)	108 (63.9)	267 (60.7)	
Yes	286 (39.6)	52 (46.0)	61 (36.1)	173 (39.3)	
SDS: Have your symptoms disrupted your family life/home responsibilities? N (%)					
Count	722	113	169	440	0.1290
No	447 (61.9)	61 (54.0)	111 (65.7)	275 (62.5)	
Yes	275 (38.1)	52 (46.0)	58 (34.3)	165 (37.5)	

Characteristics of the allergic rhinitis (AR) population. Disease severity based on the Allergic Rhinitis and its Impact on Asthma (ARIA) classification; control of AR measured by the Rhinitis Control Assessment Test (RCAT); Sleepiness measured by the Epworth Sleepiness Scale (ESS). A modified version of the Sheehan Disability Scale (SDS) was used to assess the impact on daily activities. The *p* values were calculated using the X^2^ test

The SDS was used with the permission of the SDS copyright holder Professor DV Sheehan. © Sheehan DV 1996 & 2008 & 2016

### Impact of allergic rhinitis on quality of life

**Fig. 3:**
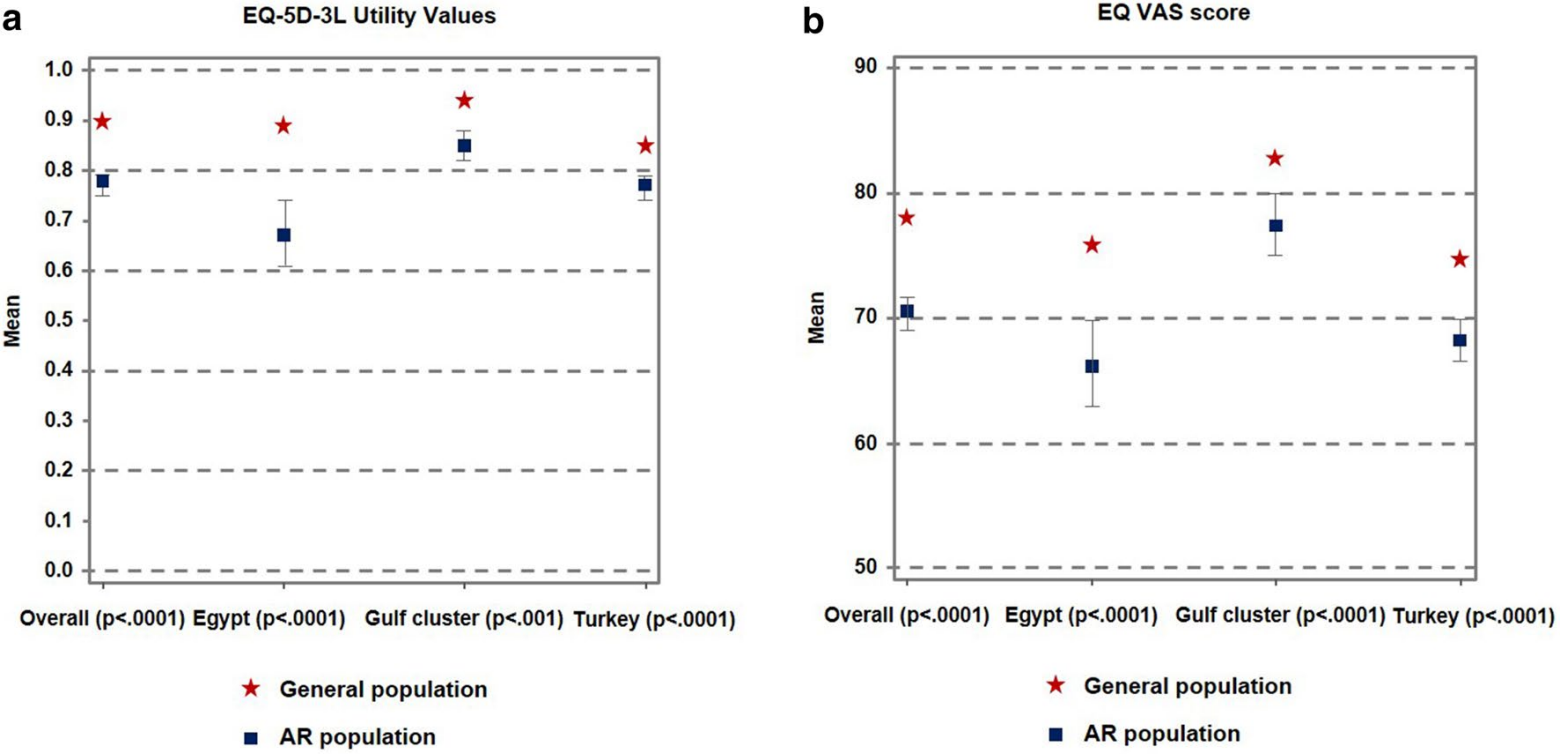
Impact of allergic rhinitis on quality of life. Quality of life was assessed using the three level EuroQol Five Dimension questionnaire (EQ-5D-3L) **a** EQ-5D-3L utility values for the allergic rhinitis (AR) population and general population by country **b** EQ-VAS scores for the AR population and general population by country. For the AR population (n = 1808) the data represent the mean EQ-5D-3L utility value and EQ-VAS score with the 95% CI. For the general population (n = 33,486), the mean EQ-5D-3L utility value and EQ-VAS score is presented. The *p* values were calculated using the Kruskall–Wallis test. Permission to use the EQ-5D-3L was provided by the EuroQol Research Foundation (© EuroQol Research Foundation. EQ-5D™ is a trade mark of the EuroQol Research Foundation)

Overall, subjects with AR reported a significantly lower (*p* < 0.0001) mean EQ-5D-3L utility score (0.78 ± 0.32) than the general population (0.90 ± 0.21). This relationship was observed for all participating countries or cluster of countries (Fig. 3a). A similar observation was made for the mean EQ-VAS scores; 70.4 ± 20.0 in subjects with AR compared to 78.1 ± 17.5 in the general population (*p *< 0.0001). The overall impact and the country-level results are presented in Fig. 3b.

### Impact of allergic rhinitis control and disease severity on quality of life

Subjects with AR whose symptoms were well-controlled reported a significantly higher EQ-5D-3L utility value (0.9 ± 0.2) and EQ-VAS score (73.4 ± 19.3) compared to those with symptoms that were not well-controlled (0.7 ± 0.4 and 67.1 ± 19.6) (*p *< 0.0001). This trend was the same across each country and the cluster of countries studied. Those subjects with moderate/severe AR reported significantly lower EQ-5D-3L utility values (0.74 ± 0.32) and EQ-VAS scores (68.4 ± 20.6) than those with mild disease (0.87 ± 0.24 0 and 74.8 ± 17.8 respectively) (*p *< 0.0001). This trend was the same across each country and the cluster of countries studied.

### Impact of allergic rhinitis control and disease severity on activities of daily living

**Fig. 4:**
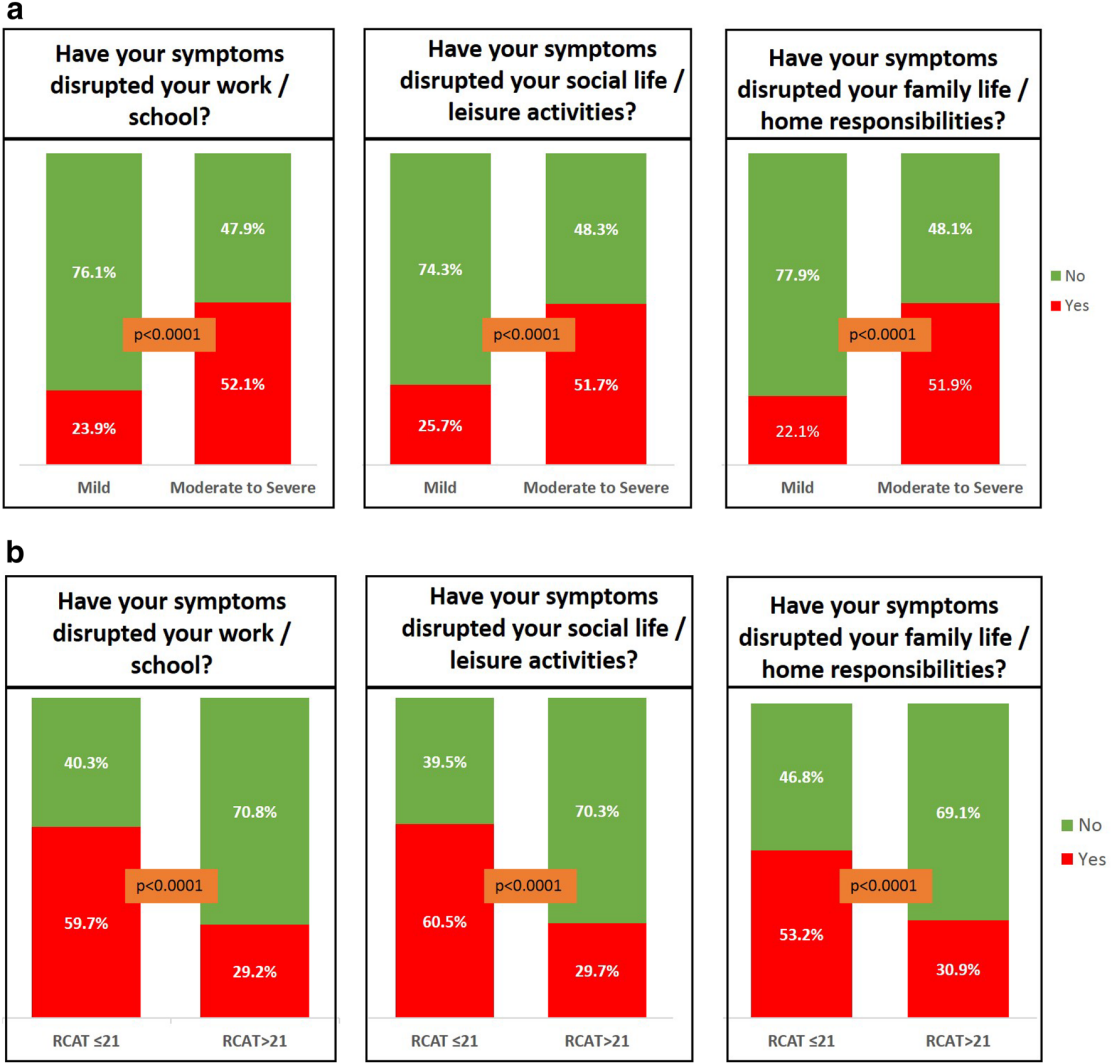
Impact of allergic rhinitis control and disease severity on daily life. Impact of allergic rhinitis control and disease severity on activities of daily living **a** impact of disease severity based on the allergic rhinitis and its impact on asthma (ARIA) classification on activities of daily living, assessed using the Sheehan Disability Scale (SDS) **b** impact of AR control measured using the Rhinitis Control Assessment Test (RCAT) on activities of daily living measured using the SDS. The *p* values were calculated using the X^2^ test

Across all dimensions of daily living a higher proportion of those with moderate/severe disease reported an impact compared to those with mild disease (*p *< 0.0001); see Fig. 4a. In addition, across all dimensions of daily living a higher proportion of those with uncontrolled symptoms (RCAT ≤ 21) reported an impact compared to those with symptoms that were well controlled (RCAT > 21) (*p *< 0.0001); see Fig. 4b.

### Impact of allergic rhinitis control on daytime sleepiness

**Fig. 5:**
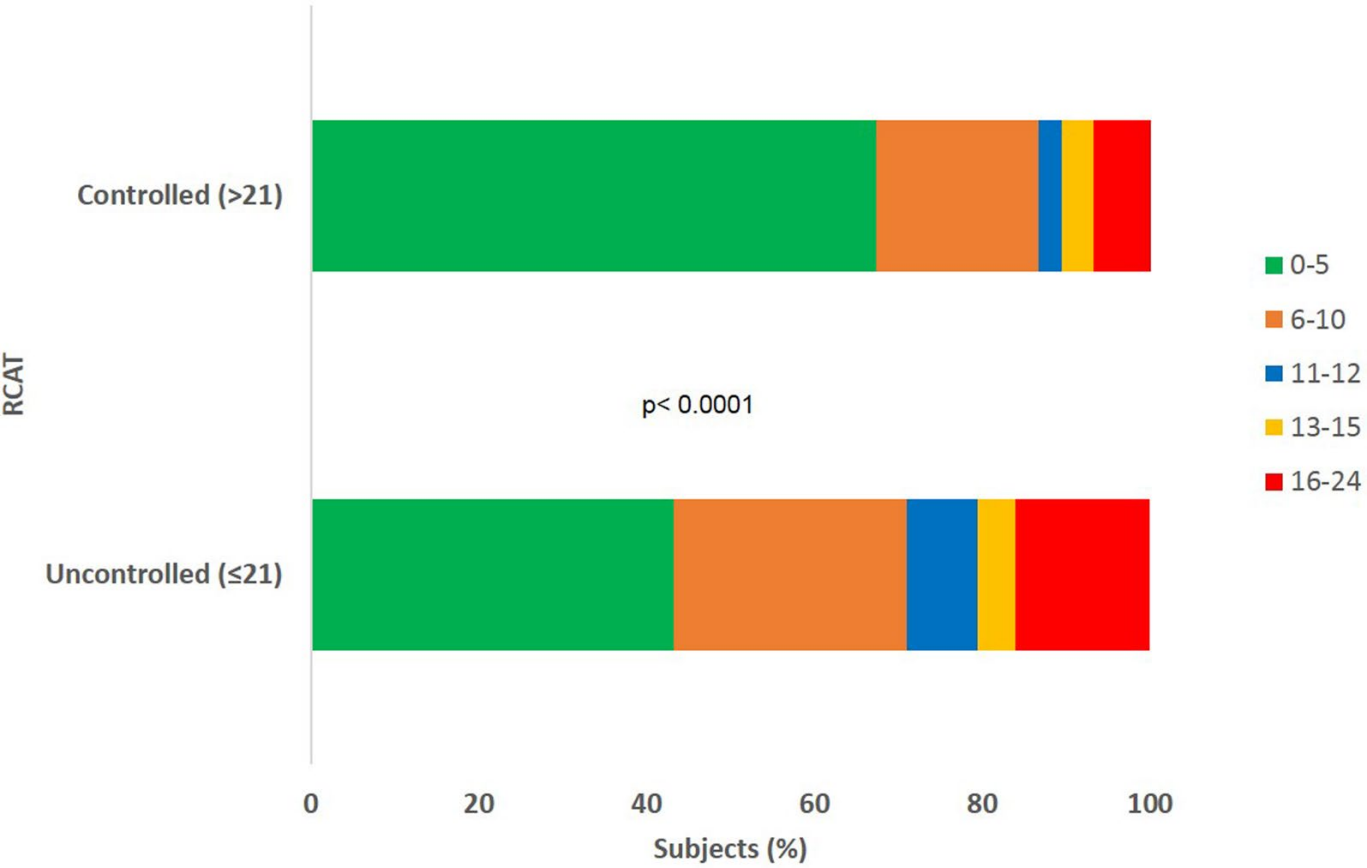
Impact of allergic rhinitis control on daytime sleepiness. Impact of AR control measured using the Rhinitis Control Assessment Test (RCAT) on reported levels of daytime sleepiness, measured by the Epworth Sleepiness Scale (ESS). The *p* value was calculated using the X^2^ test

A higher proportion of subjects with uncontrolled symptoms (27.3%) reported severe daytime sleepiness (ESS 16–24) compared to those with well-controlled symptoms (16.3%) (*p *< 0.0001); see Fig. 5.

## Discussion

This study reports a considerable burden of disease attributable to AR in the five countries studied. Subjects with AR reported a significant impact on activities of daily living and a negative impact on quality of life, both of which were exacerbated in severe or not well-controlled subjects. The prevalence of AR was found to be relatively low compared to that reported elsewhere. The lower prevalence of AR, compared to figures reported in Europe and the US [7, 8], could be due to many factors, such as cultural differences between the countries taking part. In addition, due to environmental differences between countries, subjects in the Middle East are likely to be exposed to different allergens, which may influence the reported prevalence.

Overall 1808 subjects screened positively for AR. Of the overall AR population, 47.4% (n = 857) completed the detailed questionnaire which collected data on disease management and were termed the AR responders. Demographically, there were few major differences between the AR responders and the overall AR population. The distribution in terms of age, gender, BMI, and smoking status is similar between the two populations. However, differences are apparent with respect to health system coverage. A higher proportion of subjects in the AR responders group are covered by social security and a lower proportion are uninsured. This difference is probably due to the relative contribution of the three countries/cluster involved, which have significant differences in terms of the healthcare coverage available. In Turkey, most of the population (90%) has healthcare coverage through a social security system, while in the Gulf cluster healthcare coverage is expanding through medical insurance as increasingly more individuals receive healthcare in a reimbursed manner. In Egypt, for much of the population, healthcare is paid by patients out of pocket. The response rate in the three countries/cluster differed. There is a higher percentage of Turkish subjects in the AR responder population (56%) compared to the overall AR population (34%), which is reflected in the higher proportion of AR responders covered by social security compared to the overall AR population. Given the potential impact of this on the parameters associated with disease management outcomes, such as quality of life, severity and control, these results have been presented by country.

The SFAR questionnaire is a validated self-report measure, which considers most features of the disease (clinical symptoms, seasonality, family history, medical history and perceived allergy) to screen for AR, and is considered to have a higher level of sensitivity and specificity than many other questionnaires used to screen for the disease, such as that used in the European Community Respiratory Health Survey (ECHRS). However, it is possible that in this study, the first question of the SFAR questionnaire excluded many subjects due to unawareness of AR as a disease in the Middle East. Many subjects, particularly those with mild symptoms, in the absence of a physician diagnosis of AR, might interpret their recurrent nasal symptoms as being due to a cold or sinus disease and answer no to the first SFAR question and be excluded from the rest of the questionnaire. In line with this, almost 60% of AR subjects in SNAPSHOT have ARIA defined moderate/severe disease (approximately three quarters of subjects in Egypt and the Gulf cluster) suggesting that many subjects with mild disease may have been missed due to unawareness of the disease. In other countries in Europe and North America, where the SFAR questionnaire has been used widely, and awareness of AR is higher, the first question is necessary to eliminate subjects who would otherwise be wrongly classified. Moving forward, a modified version of the SFAR, may be needed in the Middle East to account for the cultural differences in the region and avoid missing subjects who would otherwise screen positive for the disease. It should also be noted that the SFAR can assess AR when the disease is not symptomatic. It is possible to reach a score of six or seven points from questions other than those on nasal symptoms. Although this does not happen frequently, it has been observed and can reduce the possibility of misclassification of subjects. Although the reported prevalence estimates from SNAPSHOT are low, the individuals identified are likely to represent the more severe end of the spectrum where the impact of AR is greatest and where medical intervention is most needed. In addition, some subjects may already be taking medications for AR. These subjects may not have experienced AR nasal symptoms in the past 12 months due to an effective treatment strategy, and therefore answered no to question one of the SFAR. Thus, although question eight asks about a previous physician diagnosis of AR, some subjects with well-treated physician diagnosed AR and therefore not experiencing bothersome symptoms, may have been excluded from the AR population, contributing to the lower prevalence estimate observed. Therefore, the estimates provided on AR prevalence and its symptoms in the Middle East in the SNAPSHOT study can be considered as conservative. For future studies on AR in the Middle East, it could be considered to first ask questions about the physician confirmed AR diagnosis and medication usage since the dates of diagnosis, followed up by questions about the AR symptoms. This will enable a chronological view of the AR diagnosis, disease management, and patient journey to be obtained which can help in better understanding the level of AR prevalence and symptoms.

Limited data is available on the prevalence of AR in adults across the Middle East, and prevalence estimates vary widely both between countries and within countries. For example, one study carried out across all seven Emirates of the UAE used the ECRHS to screen for AR, and reported that at least 7% of the study population had the disease [9]. Another study conducted in Al Ain city in the UAE used a modified version of the ISAAC questionnaire, and reported a much higher prevalence at 32% [26]. Many factors are likely to contribute to this difference; such as the case definition and study methodology used, the demographics of the respondents, and the rural versus urban split of the population.

AIMES is the only multi-country study to have been conducted in the Middle East, which was carried out in five countries (Egypt, Iran, Lebanon, Saudi Arabia and the UAE) and reported the overall prevalence of physician-diagnosed AR to be 10%, ranging from 8% in Lebanon to 11% in Egypt [27]. The prevalence of AR reported in AIMES is higher than SNAPSHOT, however, there were methodological differences in this study that may have contributed this finding. In AIMES the study was not restricted to adults; all subjects ≥ 4 years old were eligible to participate. In addition, AIMES used a custom-designed questionnaire where a positive screening was based on a physician diagnosis of AR and experiencing symptoms and/or receiving treatment in the last 12 months. It was also mentioned that during the survey other terms that could be used to describe AR were also provided to the subject, (since it was thought that AR may not have been a familiar term in the countries where the interviews took place), to ensure that the survey captured all subjects with the condition. As already discussed, in SNAPSHOT the lack of awareness about AR as a condition, may have contributed to the low prevalence observed.

A variety of studies have been conducted in adults in Turkey. Two studies have been conducted using the ECRHS questionnaire. In Manisa, the reported AR prevalence in adults was 14.5% [10]. In a separate study in Antalya the reported prevalence was 27.7% [11]. The Prevalence And Risk Factors of Allergies In Turkey (PARFAIT) study investigated the prevalence of AR across 14 cities in Turkey and reported the prevalence of AR in males to be 11.7% in urban areas and 17.5% in rural areas, compared to 17.0% and 21.2% respectively for females [12]. Two separate population-based studies have been carried out in adults in Turkey, across seven geographical regions of the country. One study used a custom-designed questionnaire focused mainly on descriptive parameters and based the definition of AR on the ARIA guidelines. The reported prevalence from this study ranged from 16.3% in the Eastern Anatolia region to 27.5% in the Marmara region [28]. The second study used the SFAR questionnaire and reported an overall self-reported prevalence of 29.6% for the whole study group. In line with the previous study, the highest prevalence of self-reported AR was reported in the Marmara region (36.1%) and the lowest was reported in South East Anatolia region (21%) [13]. The only other study in the Middle East which used the SFAR questionnaire was carried out in Aydin and reported a 12-month prevalence of AR in adults of 14% [29]. These differences in prevalence may be due to many factors, such as differences in population selection, the characteristics of the population, the period of the year in which the survey was conducted or environmental factors. Without a like for like comparison of case definition it is difficult to compare.

Control of AR is essentially seen as absence of symptoms and is an important measure which few previous studies in the Middle East have addressed. The AIMES survey reported that less than half (40%) of respondents felt their symptoms were completely or well-controlled, and 15% described their symptoms as poorly or not controlled, despite the majority taking medication to treat their symptoms [27]. In contrast, the majority of subjects with AR in the SNAPSHOT program (67%) reported their symptoms were controlled. This could be due to the different methods used to measure control in the two studies, patient perception in the AIMES study versus the RCAT in the SNAPSHOT program.

It is well established that AR has a profound effect on quality of life, having a significant impact on activities such as sleep [2, 30], work, and social life [31]. Two-thirds of patients who took part in the Allergies in America survey, which included 2500 adults aged 18 years or older who had been diagnosed with AR, nasal allergies or hay fever and were symptomatic or had been treated for nasal allergies within the last 12 months reported that AR affected their daily life [32]. Regional data from the AIMES study reported that 58% of respondents felt their daily life was impacted by the disease; 72% of respondents reported their performance at work/school was affected and 80% reported that AR impacted their sleep [27]. The SNAPSHOT results are in line with the literature; subjects with AR reported a significant impact on quality of life across all countries and the cluster of countries studied, which was amplified in more severe disease and disease that was not controlled, which is also supported in the literature. For example, a French study in 3052 patients consulting general practitioners for AR investigated the effects of severity and duration (intermittent or persistent) of AR on impairment of quality of life, sleep and work performance. Disease severity had more of an effect than duration and the majority (80%) of those with moderate/severe disease reported impaired activities, compared to only 40% of those with mild symptoms [33].

The impact of AR on daily activities was assessed using the SDS. The SDS was modified for use in this program from its usual 0 to 10 point discretized analogue layout anchored both numerically, verbally descriptively and visually spatially into 5 response option categories (a score of 0 = not at all, 1–3 = mildly, 4–6 = moderately, 7–9 = markedly, 10 = extremely). Accordingly, data analysis was discussed with the scale author to ensure correct interpretation of the results. The use of the 12-month time frame in this study enables the data to be more relevant for governments and healthcare bodies to aid in developing healthcare policy. The questionnaire has been validated for use in face-to-face interviews, but was administered by telephone in SNAPSHOT, which should not affect the validity of the data collected.

It has frequently been reported that AR results in increased daytime sleepiness. For example, a clinical trial comparing 25 healthy individuals with 25 people with seasonal AR found a significant increase in daytime sleepiness reported by those with AR and these patients also reported significant impairment to their quality of life. The impact was related to the severity of the disease [34]. Interestingly, data from SNAPSHOT shows that most subjects experience low levels of daytime sleepiness, despite over half describing their symptoms as moderate/severe. However, this may be a consequence of most subjects reporting symptom control.

As with all studies, limitations do exist. These include the fact the survey was telephone based, which could introduce a sampling bias if certain groups do not have access to a telephone, such as those in more remote, rural areas. However, the SNAPSHOT program considered both mobile and fixed landline telephone numbers to be eligible, and the emergence of mobile telephones means that most households in these countries have access to a telephone. The survey was conducted by trained lay interviewers; and the case definition is based on perceived symptoms with no physician confirmation. However, the clinical definition of AR is difficult to implement in an epidemiological setting where large populations are studied, since it is not possible to obtain laboratory evidence of an immune response. As is the case for all studies that require participants to recall data, there is a risk of inaccuracy in the data collected. In addition, as explained in detail earlier, the interpretation of the first question of the SFAR in this region is likely to have excluded a number of subjects with AR from the program resulting in a potential underestimation of the prevalence of AR in the countries studied. It is important to collect data on symptoms of AR in such countries, since awareness of AR as a disease is low in the region.

## Conclusion

This study provides an updated prevalence estimate for AR and information on the disease burden within the adult general population of five countries in the Middle East region, using an identical methodology across all countries studied. Although the observed prevalence of AR in these Middle Eastern countries is lower than that reported in western countries, its burden is considerable. AR, and specifically uncontrolled and severe disease results in a negative impact on quality of life, quality of sleep and daily activities. The results from this study can contribute to informed decision making when setting priorities for public health policy and strategy to manage AR in these countries.

## Abbreviations

AIMES: Allergies in the Middle East Survey
AIR: Asthma Insights and Reality
AR: Allergic Rhinitis
ARIA: Allergic Rhinitis and its Impact on Asthma
BMI: Body Mass Index
BPH: Benign Prostatic Hyperplasia
CAPI: Computer-Assisted Personal Interviewing
CI: Confidence Interval
COPD: Chronic Obstructive Pulmonary Disease
CRO: Contract Research Organisation
ECRHS: European Community Respiratory Health Survey
EQ-5D: EuroQoL Five Dimension questionnaire
EQ-5D-3L: 3 Level EQ-5D questionnaire
EQ-VAS: EuroQol Visual Analogue Scale
ESS: Epworth Sleepiness Scale
GEP: Good Epidemiological Practice
HTA: Health Technology Assessment
ISAAC: International Study of Asthma and Allergies in Childhood
IQR: Inter Quartile Range
OR: Odds Ratio
PARFAIT: Prevalence and Risk Factors of Allergies in Turkey
RCAT: Rhinitis Control Assessment Test
SD: Standard Deviation
SDS: Sheehan Disability Scale
SFAR: Score For Allergic Rhinitis
SFDA: Saudi Food and Drug Authority
UAE: United Arab Emirates
UK: United Kingdom
US: United States
WHO: World Health Organisation
